# A New Departure

**Published:** 1889-11

**Authors:** 


### EDITORIALS AND MISCELLANEOUS.

A NEW DEPARTURE.—It is proper to say to our readers that new and important changes are in contemplation for this Journal, to commence with the January No. 1889. The times are progressive, and all who wish to succeed must keep up with them.

Arrangements are not yet sufficiently perfected to enable us to give the new programme in this issue. This much however, we will state as fully determined upon :

A new and active Business Manager will take the place of Dr. Word, who finds it necessary to lay off a portion of the heavy and accumulating duties, which, in connection with his practice and college labors, have become too burdensome for his health and advancing years.

The Journal will be brought out in somewhat different style. Eight new pages of reading matter will be added, and other changes made looking to a higher and more practical standard of excellence and usefulness in all of its departments; so that it shall become more than ever the favorite of the busy practitioner, and the exponent of medical literature in the Southern States.

To this end it will be necessary that all old accounts for subscription falling due at the close of the present year be settled as soon as possible with the present business manager. We earnestly hope that our friends will be prompt in this matter, and that each one will not only renew his subscription, but send us new names for our list, thus enabling us to carry out more effectually the schemes for improvement which will enure to the benefit of our readers not less than to the Journal itself.

